# Comparison of Ginsenoside Components of Various Tissues of New Zealand Forest-Grown Asian Ginseng (*Panax Ginseng*) and American Ginseng (*Panax Quinquefolium* L.)

**DOI:** 10.3390/biom10030372

**Published:** 2020-02-28

**Authors:** Wei Chen, Prabhu Balan, David G Popovich

**Affiliations:** 1 School of Food and Advanced Technology, Massey University, Palmerston North 4442, New Zealand; w.chen2@massey.ac.nz; 2 Riddet Institute, Massey University, Palmerston North 4442, New Zealand; p.balan@massey.ac.nz; 3 Alpha-Massey Natural Nutraceutical Research Centre, Massey University, Palmerston North 4442, New Zealand

**Keywords:** Asian ginseng, American ginseng, *Panax ginseng*, *Panax quinquefolium* L., ginsenosides

## Abstract

Asian ginseng (*Panax ginseng*) and American ginseng (*Panax quinquefolium* L.) are the two most important ginseng species for their medicinal properties. Ginseng is not only popular to consume, but is also increasingly popular to cultivate. In the North Island of New Zealand, Asian ginseng and American ginseng have been grown in Taupo and Rotorua for more than 15 years. There are no publications comparing the chemical constituents between New Zealand-grown Asian ginseng (NZPG) and New Zealand-grown American ginseng (NZPQ). In this study, fourteen ginsenoside reference standards and LC–MS^2^ technology were employed to analyze the ginsenoside components of various parts (fine root, rhizome, main root, stem, and leaf) from NZPG and NZPQ. Fifty and 43 ginsenosides were identified from various parts of NZPG and NZPQ, respectively, and 29 ginsenosides were found in both ginseng species. Ginsenoside concentrations in different parts of ginsengs were varied. Compared to other tissues, the fine roots contained the most abundant ginsenosides, not only in NZPG (142.49 ± 1.14 mg/g) but also in NZPQ (115.69 ± 3.51 mg/g). For the individual ginsenosides of both NZPG and NZPQ, concentration of Rb1 was highest in the underground parts (fine root, rhizome, and main root), and ginsenoside Re was highest in the aboveground parts (stem and leaf).

## 1. Introduction

Ginseng is a perennial herb and belongs to the genus *Panax* (Araliaceae family). There are more than twelve species of ginseng characterized in the genus *Panax*. Among them, Asian ginseng and American ginseng are the two most well-known species and are commonly used for their medicinal properties [1]. Asian ginseng (*Panax ginseng*) root has been used widely as a significant source of natural medicine for thousands of years in East Asia, particularly in China, Korea, and Japan. American ginseng (*Panax quinquefolium* L.), mainly grown in North America, is also well-known in Asian countries. As important Chinese medicine resources, Asian ginseng and American ginseng are considered to possess different properties in clinical applications. Asian ginseng, especially Korean red ginseng, has a warming effect and reinforces ‘qi’ to promote ‘yang’ energy, which benefits the spleen and lungs [2], and it was found to modulate the intestinal ecosystem and enhance the gut function [3]. American ginseng is viewed as having a cooling effect and can tonify ‘qi’ to nourish ‘yin’ energy, which means it can remove ’heat’ and promote the production of body fluids [4]. These functional varieties may be due to differences in chemical composition, particularly in the bioactive triterpenoid saponins, popularly known as ginsenosides [5].

Ginsenosides, also known as triterpenoid saponins with a four-ring skeleton structure, are unique to ginseng species. So far, nearly 200 ginsenosides have been identified in Asian ginseng, and more than 100 in American ginseng; 49 ginsenosides coexist in both plant species [6]. Most of them are classified as members of the dammarane family, including the protopanaxadiol (PPD) type and protopanaxatriol (PPT) type. In chemical analysis of ginseng, ginsenosides Rb1, Rb2, Rb3, Rc, Rd, Re, Rf, Rg1, and p-F11 are the most important compounds to be quantified due to their abundant content in ginseng plants. Among these compounds, ginsenoside Rf is considered to be only present in Asian ginseng, while ginsenoside p-F11 is considered to be only found in American ginseng [7]. Therefore, ginsenosides Rf and p-F11 are used as marker compounds to differentiate Asian ginseng and American ginseng [5,8]. There are some articles about the component analysis of Asian ginseng and American ginseng; they mainly focus on how to differentiate the two ginseng species [9] or the constituent analysis of ginseng roots [10,11]. To our knowledge, there have been no comprehensive analyses of the ginsenoside components of ginseng tissues (fine root, rhizome, main root, stem, and leaf) between Asian ginseng and American ginseng. Furthermore, the vast majority of ginseng used in ginseng studies has been grown in the Northern Hemisphere; there are few reports about how ginseng grows in the Southern Hemisphere, such as those varieties in New Zealand.

New Zealand has a unique geographical environment, with features such as cold winters, temperate summers, adequate rainfall, unique volcanic pumice soil, and high-intensity UV rays. Summer solar radiation in New Zealand is on average 7% higher compared to an equivalent latitude in the Northern Hemisphere [12]. These factors may affect the accumulation and distribution of ginsenosides in ginseng plants. Our previous study found that the average content of total ginsenosides from New Zealand-grown *Panax ginseng* is significantly higher than that of ginseng grown in China or Korea [13]. In this study, we used LC–MS^2^ technology to analyze the ginsenoside components of various parts from New Zealand-grown Asian ginseng (NZPG) and New Zealand-grown American ginseng (NZPQ). To our knowledge, this is the first article focusing on ginsenoside component analysis of ginseng tissues between Asian ginseng and American ginseng, particularly for New Zealand-grown ginseng.

## 2. Materials and Methods

### 2.1. Ginseng Samples

Eight-year-old Asian ginseng and American ginseng plants were harvested in November 2017 from a Taupo (New Zealand) pine forest. Five parts including fine root (root hair), rhizome (neck between root and stem), main root (root body), stem, and leaf of Asian ginseng and American ginseng were separated, rinsed with water, and lyophilized at –68 ℃. The dried ginseng tissues were then powdered using a CG2B coffee grinder (Breville).

### 2.2. Chemicals and Reagents

**Figure 1:**
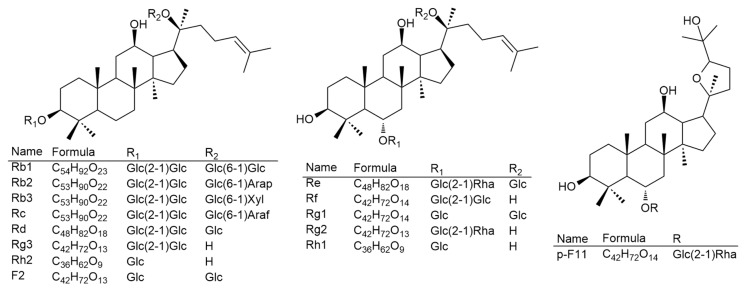
The chemical structures of 14 ginsenoside reference standards.

Fourteen reference standards of ginsenosides Rb1, Rb2, Rb3, Rc, Rd, Re, Rf, Rg1, Rg2, Rg3, Rh1, Rh2, F2, and 24(R)-pseudoginsenoside F11 (p-F11) were purchased from Star Ocean Ginseng Ltd. (Suzhou, China). The purities of the fourteen standards were no less than 98.0%. Their structures are shown in Figure 1. LC–MS-grade acetonitrile (MeCN) and water were supplied by Merck (Phillipsburg, NJ, USA). LC-grade methanol (MeOH) and formic acid (HCOOH) were purchased from Fisher Chemical (Pittsburgh, PA, USA). Water (for extraction) was obtained from a Milli-Q Ultra-pure water system (Millipore, Billerica, MA, USA). Other reagents used in this study were of analytical grade.

### 2.3. Preparation of Samples and Reference Standards

Ginsenosides were ultrasonically extracted three times from each part of the Asian ginseng and American ginseng using a Q700 sonicator (Qsonica, Melville, NY, USA) according to our previous methods [13,14]. Briefly, the fine root, rhizome, main root, stem, and leaf were separately extracted by 70% (*v/v*) aqueous MeOH at 20 kHz for 10 min at no more than 40 ℃. (The extraction was programed for five cycles; each cycle contained ultrasonic extraction at 15% amplitude for 2 min and a cooling period of 1 min between extractions.) The supernatant was collected after centrifugation at 4000 rpm (Thermo Scientific Multifuge 1S-R Centrifuge, Marshall Scientific, Hampton, NH, USA) for 10 min and the sediment was extracted twice more. The three extracts were mixed together and filtered through a 0.22-micron syringe filter before the LC/MS analysis. Fourteen reference standards of ginsenosides Rb1 (0.769 mg/mL), Rb2 (0.846 mg/mL), Rb3 (0.629 mg/mL), Rc (1.077 mg/mL), Rd (0.692 mg/mL), Re (0.923 mg/mL), Rf (1.462 mg/mL), F2 (0.692 mg/mL), p-F11 (0.923 mg/mL), Rg1 (1.154 mg/mL), Rg2 (0.615 mg/mL), Rg3 (1.154 mg/mL), Rh1 (1.000 mg/mL), and Rh2 (1.077 mg/mL) were dissolved and diluted with 70% MeOH to obtain a series of standard solutions of different concentrations. The solutions were filtered through a 0.22-micron syringe filter before the LC–MS^2^ analysis.

### 2.4. High-Performance Liquid Chromatography Coupled with Quadrupole Time-of-Flight Tandem Mass Spectrometry (HPLC-QTOF-MS)

An Agilent 1290 liquid chromatograph (Agilent, Lexington, MA, USA) equipped with an online degasser, a quaternary pump, an auto-sampler, a heated column compartment, a UV detector, and an Agilent 6530 Quadrupole Time-of-Flight Mass Spectrometer (Agilent, Lexington, MA, USA) equipped with an electrospray ionization source were used for LC–MS^2^ analysis. The instrument setting was consistent with our previous reports [13,14]. A double end-capped Zorbax Extend-C18 (2.1 × 100 mm, 3.5 μm) column (Agilent, Lexington, MA, USA) was used to separate compounds from the ginseng extract. The column temperature was controlled at 33 ℃. The binary gradient eluent consisted of mobile phase A (0.1% formic acid in water) and mobile phase B (0.1% formic acid in acetonitrile). The gradient elution program was as follows: 20% B at 0–4 min, 20–30% B at 6–10 min, 30–32.5% B at 10–25 min, 32.5–60% B at 25–27 min, 60–95% B at 27–39 min, 95% B at 39–40 min. The flow rate was changed with the gradient: 0–27 min, 0.2 mL/min; 27–40 min, 0.25 mL/min. The wavelength was set at 203 nm, and the injected volume was 1 μL. The mass spectrometer data were collected from *m/z* 100–2200 in negative ion mode, and nitrogen (>99.998%) was used for the nebulizer gas and curtain gas. The gas temperature and flow rate were 350 ℃ and 10.0 L/min, respectively. The pressure of the nebulizer was 37 psi. The voltages of capillary, fragmentor, and skimmer were 3500 V, 220 V, and 65 V, respectively. The reference masses in negative ion mode were at *m/z* 121.0509 and 922.0098. The acquisition rates were 4 spectra/s for MS and 1 spectrum/s for MS^2^. Mass data were analyzed with Agilent Mass Hunter Workstation software (version B.06.00; Agilent Technologies, Santa Clara, CA, USA).

## 3. Results and Discussion

### 3.1. Identification of the Detected Ginsenosides in Various Parts of NZPG and NZPQ

**Figure 2:**
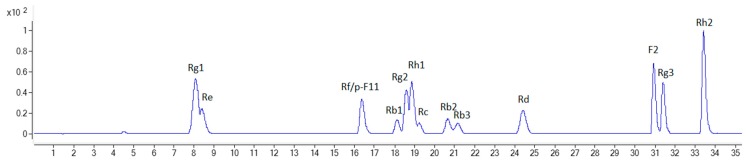
The base peak chromatogram (BPC) of 14 ginsenoside reference standards.

**Figure 3:**
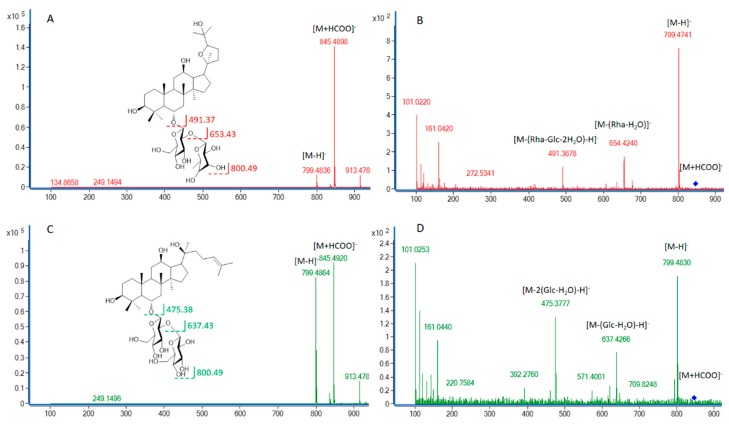
The MS (**A**, **C**) and MS^2^ (**B**, **D**) of ginsenosides p-F11 (**A**, **B**) and Rf (**C**, **D**).

To compare the ginsenoside components from various parts of the ginseng plants, Zorbax Extend-C18 column and LC-QTOF-MS/MS were used to separate and determine the ginsenosides extracted from the fine root, rhizome, main root, stem, and leaf of NZPG and NZPQ. Fourteen ginsenosides reference standards (for structures see Figure 1) were used to establish the chromatographic method. The base peak chromatograph (BPC) of the ginsenoside standards is shown in Figure 2. Although ginsenosides Rf and p-F11 are isomeric compounds and have the same retention time, we can differentiate them using mass spectrometry (MS). As shown in Figure 3A,C, ginsenosides Rf and p-F11 had similar [M - H]^−^ ion at *m/z* 799.48 and [M + HCOO]^−^ ion at *m/z* 845.49 in the negative ESI full scan MS. The abundance of *m/z* 799.48 [M - H]^−^ and *m/z* 845.49 [M + HCOO]^−^ are almost equal in ginsenoside Rf, while in ginsenoside p-F11, the abundance of *m/z* 845.59 [M + HCOO]^−^ is about seven times that of *m/z* 799.48 [M - H]^−^. In the MS^2^, ginsenosides Rf and p-F11 have the same [M - H]^−^ ion at *m/z* 799.48 and distinct product ions at *m/z* (637.42, 475.38) and (654.42, 491.37), respectively, which were obtained by the successive losses of the two sugar moieties (shown in Figure 3B,D). These distinct ions could be used as the characteristic ions to differentiate and quantify the two ginsenoside isomers.

**Figure 4:**
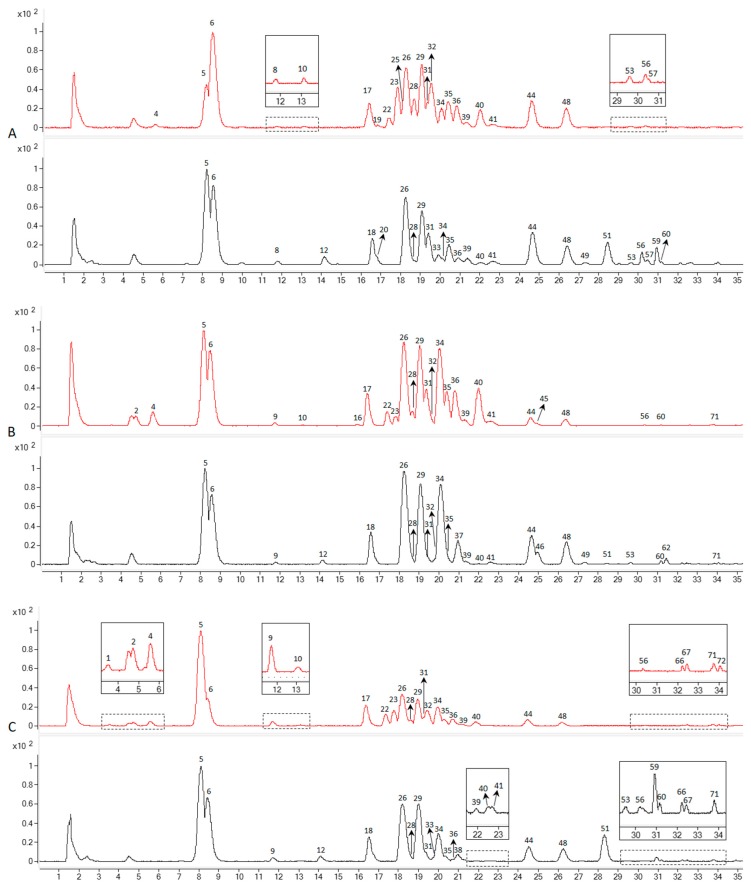

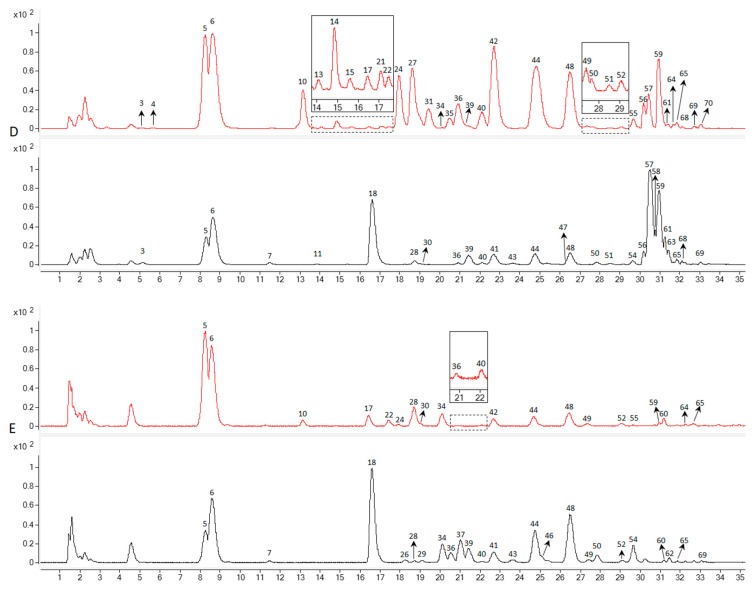
The base peak chromatogram (BPC) profiles of different parts of New Zealand-grown Asian ginseng (NZPG, red line) and New Zealand-grown American ginseng (NZPQ, black line). (**A**) Ginseng fine root, (**B**) ginseng rhizome, (**C**) ginseng main root, (**D**) ginseng leaf, (**E**) ginseng stem.

The BPC profiles of the various parts of NZPG and NZPQ are shown in Figure 4. A total of 72 potential ginsenosides were detected from the fine root, rhizome, main root, leaf, and stem of NZPG and NZPQ. The potential ginsenosides were identified using the same procedure as our previous report [14]. Briefly, the PPD-type, PPT-type, and oleanolic acid-type ginsenosides could produce [(20S)-protopanaxadiol - H]^-^ at *m/z* 459 (C_30_H_51_O_3_), [(20S)-protopanaxatriol - H]^−^ at *m/z* 475 (C_30_H_51_O_4_), and [oleanolic acid - H]^−^ at *m/z* 455 (C_30_H_47_O_3_) in the negative MS^2^ spectra, respectively. Thus, a different type of ginsenoside could be easily differentiated and characterized by finding its [aglycone - H]^-^. Sugar residues could be identified by calculating the neutral loss molecular mass, for instance, the glucosyl (Glc), rhamnosyl (Rha), and pentosyl [arabinopyranosyl (Arap) or arabinofuranosyl (Araf) or xylosyl (Xyl)] groups correspond to the mass differences of 162, 146, and 132 amu in the MS^2^ spectra, respectively. There are some small mass differences, such as 43 and 87 amu, which are mostly acetyl and malonyl groups. These groups are prone to attach with glucose.

Fourteen ginsenosides (peaks 5, 6, 17, 18, 26, 28, 30, 31, 36, 39, 44, 59, 61, 70) were unambiguously identified by comparison with their reference standards. The other peaks were allocated by comparing the relative retention time, empirical molecular formulas, and fragmentation information with those in the literature.

**Figure 5:**
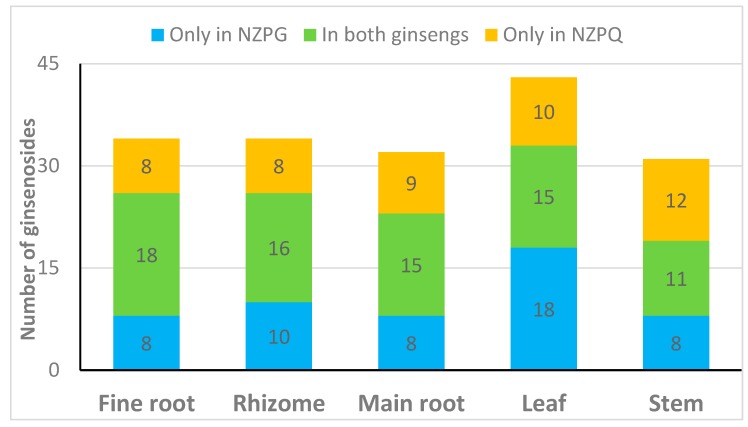
Distribution of ginsenosides in different parts of NZPG and NZPQ.

The information on 72 detected compounds is summarized in Table 1. Apart from eight unknown compounds, 64 ginsenosides were identified from NZPG and NZPQ. In the ginseng fine root, NZPG and NZPQ contain the same number (26) of ginsenosides, and 18 of them are in both ginsengs (Figure 4A). In the ginseng rhizome, there were 16 ginsenosides found in both ginseng species, and there were 10 and eight ginsenosides found only in NZPG and NZPQ respectively (Figure 4B). As the main source of medicinal material, the chemical constituents of the main root are essential for the pharmacological activity of ginseng. From Figure 4C, we can see that in addition to the common main compounds 5 (Rg1), 6 (Re), 26 (Rb1), 29 (mRb1), 34 (Ro), 44 (Rd), and 48 (mRd) in both ginseng species, NZPG and NZPQ also contain some unique secondary metabolites, such as compounds 17 (Rf), 22 (NG-R2/G-F3/G-F5), and 23 (Ra1/Ra2) in NZPG, and compounds 18 (p-F11), and 51 (Rd isomer) in NZPQ. This may be the reason why Asian ginseng and American ginseng not only have similar bioactive effects, including enhanced physical and sexual functions, general vitality, anti-stress, and anti-aging [4], but also have different medicinal applications. Compared to the underground parts (fine root, rhizome, and main root), ginseng leaf contained more abundant ginsenosides, especially in the less polar areas (Figure 4D). Specifically, 33 and 25 ginsenosides were identified from the leaves of NZPG and NZPQ, respectively; 15 of these ginsenosides were found in both species. In the aboveground parts, there were fewer ginsenosides in stems than in the leaves. Interestingly, NZPQ had more peaks than NZPG had in ginseng stems (Figure 4E), whereas in leaves, NZPG had more peaks than NZPQ. In general, 50 and 43 ginsenosides were identified from various parts of NZPG and NZPQ, respectively, and 29 ginsenosides were found in both ginseng plants. Details about the distribution of ginsenosides are shown in Figure 5.

### 3.2. Quantification of the Main Ginsenosides in Various Parts of NZPG and NZPQ

To compare the differences in the content of ginsenosides between NZPG and NZPQ, the main ginsenosides in various parts of both ginseng plants were quantified according to our previous methods [14]. Briefly, 14 ginsenosides (Rb1, Rb2, Rb3, Rc, Rd, Re, Rf, p-F11, F2, Rg1, Rg2, Rg3, Rh1, and Rh2) were accurately quantified by their own linear regression equations of standard curves (the regression equations of calibration curves, correlation coefficient, and linear ranges for the ginsenoside standards are shown in supplementary material); some ginsenosides without reference standards, such as ginsenosides mRb1 (m = malonyl), mRb2, mRb3, mRc, mRd, mRe, and mRg1, were relatively quantified by the regression equations of their corresponding neutral ginsenosides.

**Figure 6:**
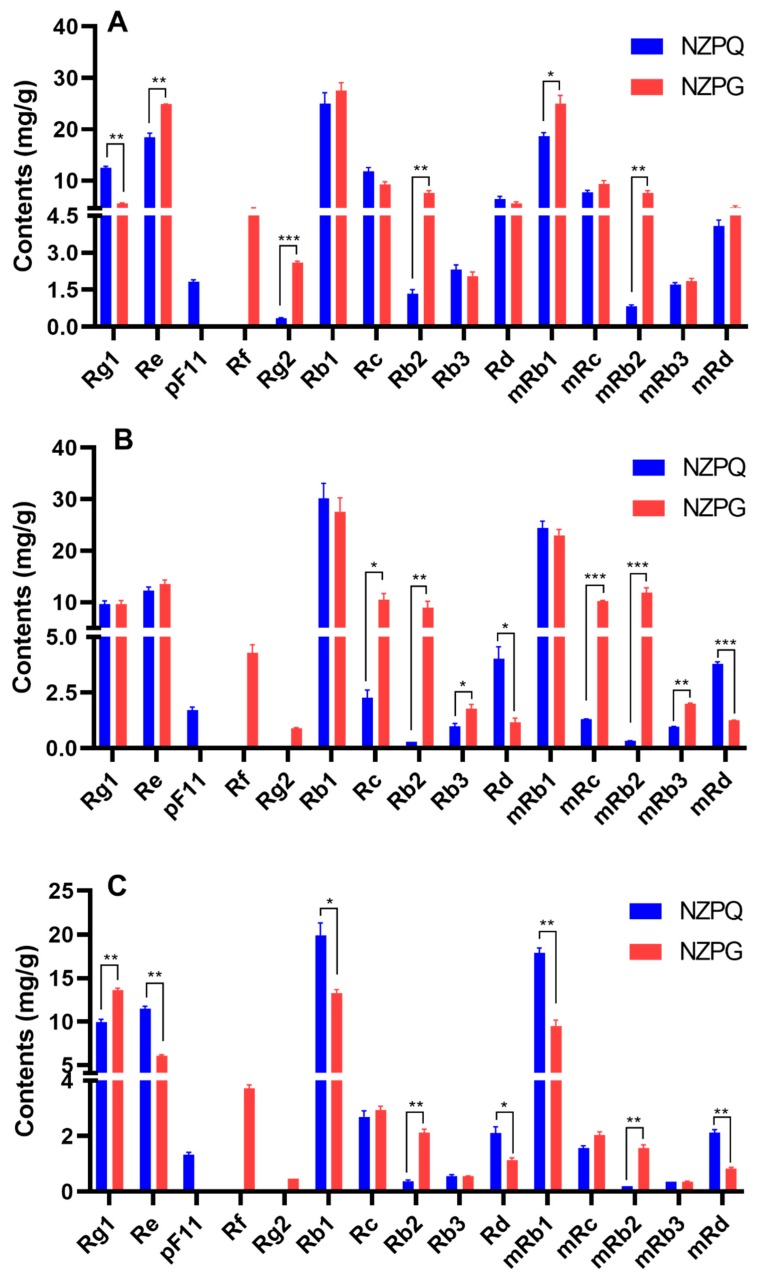

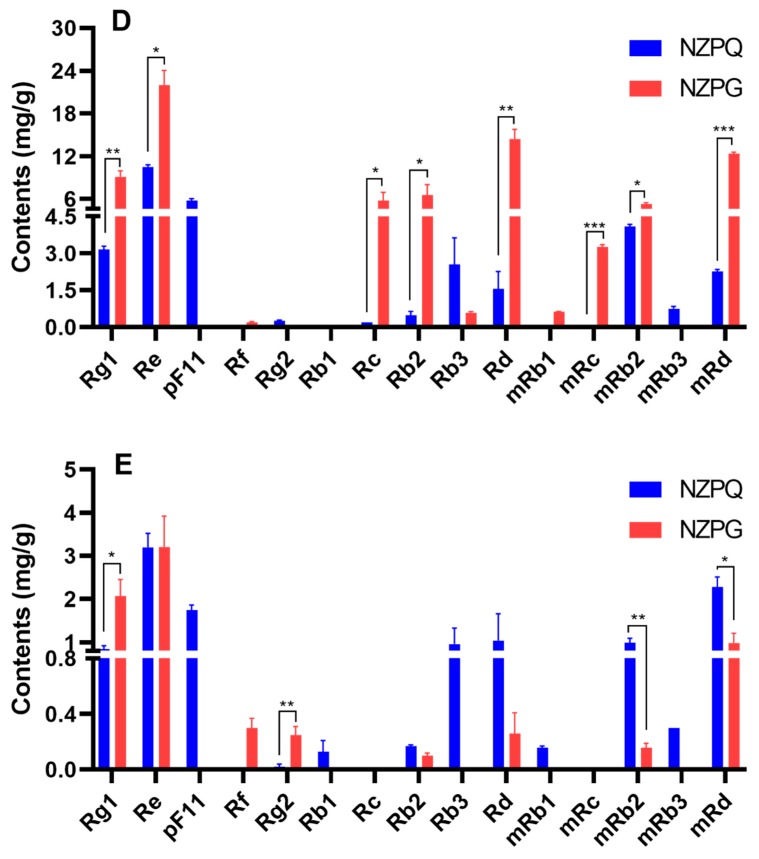
The ginsenoside contents from different parts of NZPQ and NZPG. (**A**) Fine root; (**B**) rhizome; (**C**) main root; (**D**) leaf; (**E**) stem. Data were expressed as mean ± SD and analyzed by t-test using Graph pad 8 software. * *p* < 0.05, ** *p* < 0.01, *** *p* < 0.001. Differences were considered significant if *p* < 0.05.

As shown in Figure 6, ginsenoside concentrations are varied in different ginseng tissues and different ginseng species. The four highest concentration saponins in the underground parts (fine root, rhizome, and main root) are ginsenosides Rb1, mRb1, Re, and Rg1. The ginsenoside contents differ between the two ginseng species. In the fine root (Figure 6A), ginsenoside Rb1 has the highest concentration of saponins in both NZPQ (24.95 ± 2.14 mg/g) and NZPG (27.49 ± 1.57 mg/g). The contents of ginsenosides mRb1, Re, Rg2, Rb2, and mRb2 in NZPG are significantly higher than in NZPQ. The ginsenoside Rg1 content in NZPG is significantly lower than that in NZPQ. In the rhizome (Figure 6B), ginsenosides Rb1, mRb1, Re, and Rg1 have the four highest concentration saponins, and there are no significant differences in their concentrations between NZPQ and NZPG. However, the contents of PPD-type ginsenosides Rc, Rb2, Rb3, and their corresponding malonyl ginsenosides (mRc, mRb2, and mRb3) in NZPG are significantly higher than those in NZPQ. The contents of ginsenosides Rb1, mRb1, Re, and Rd in NZPG are significantly lower than those in NZPQ in the main root (Figure 6C), and the concentrations of ginsenosides Rg1, Rb2, and mRb2 in NZPG are more than those in NZPQ. Compared to the underground parts, the distribution of ginsenosides in the aboveground parts (stem and leaf) is much different. Ginsenoside Re has the highest level of compound in the leaf and stem of both NZPG and NZPQ. Ginsenosides Rd and m-Rd became the second and third highest ingredients in NZPG leaves, while in NZPQ leaves, compound p-F11 became the major constituent, with the concentration ranking just after Re. Interestingly, ginsenosides Rb1 and mRb1, which are the main components in the underground parts, are very low and could not be quantified in some aboveground parts due to the low concentration (Figure 6D,E). As for the total ginsenoside content, the fine roots contain the most abundant ginsenosides not only in NZPG (142.49 ± 1.14 mg/g), but also in NZPQ (115.69 ± 3.51 mg/g); stems have the fewest ginsenosides. From the point of the total amount of ginsenoside, the main roots have similar concentrations between NZPG and NZPQ, while there is greater difference in the aboveground parts, especially in the ginseng leaves of both species (37.40 ± 3.35 mg/g in NZPQ vs. 91.51 ± 7.20 mg/g in NZPG).

**Figure 7:**
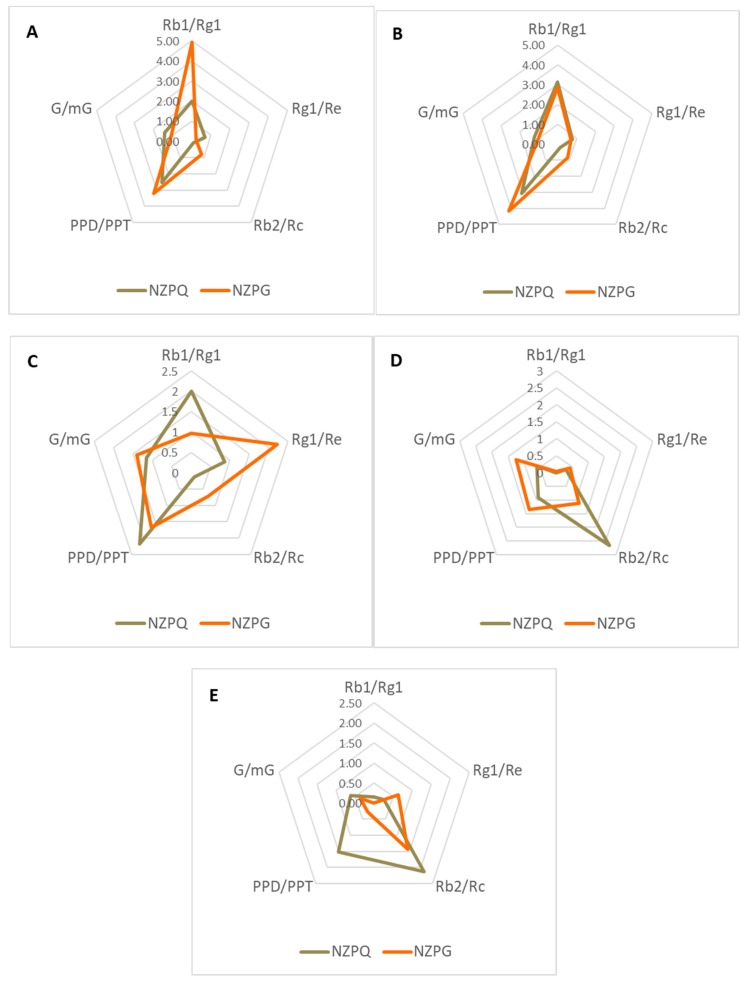
The ratios of Rb1/Rg1, Rg1/Re, Rb2/Rc, protopanaxadiol/protopanaxatriol (PPD/PPT), and G/mG in NZPQ and NZPG. (**A**) Fine root; (**B**) rhizome; (**C**) main root; (**D**) leaf; (**E**) stem. The PPD-type amount and PPT-type amounts are the sum of all the quantified PPD-type ginsenosides and PPT-type ginsenosides, respectively. G/mG is the ratio of neutral ginsenoside amount to malonyl ginsenoside amount; the malonyl ginsenoside amount is the sum of five quantified malonyl ginsenosides (mRb1, mRb2, mRb3, mRc, and mRd), and the neutral ginsenoside amount is the sum of corresponding neutral ginsenosides (Rb1, Rb2, Rb3, Rc, and Rd).

Apart from the individual ginsenoside content and the total ginsenoside amount, the ratio of PPD-type to PPT-type (PPD/PPT), the ratio of neutral ginsenoside to malonyl ginsenoside (G/m-G), and the ratios of Rb1/Rg1, Rg1/Re, and Rb2/Rc are also described in this study. These ratios in the five ginseng parts vary in both ginseng species. The Rb1/Rg1 of NZPG (4.94 ± 0.31) is much higher (*p* < 0.05) than that of NZPQ (2.01 ± 0.07) in the fine roots (Figure 7A). There are similar ratios of ginsenosides in the rhizome of both ginseng species (Figure 7B). In the main root, the Rb1/Rg1 in NZPG (0.97 ± 0.03) is significantly lower than in NZPQ (2.00 ± 0.11); the ratios of Rg1/Re and Rb2/Rc in NZPG are significantly higher than in NZPQ (Figure 7C). In the aboveground parts, NZPQ (2.67 ± 0.13) has a significantly higher ratio of Rb2/Rc compared to NZPG (1.13 ± 0.09) in the ginseng leaves (Figure 7D), and the ratio of PPD/PPT in NZPQ stems is remarkably higher than that in NZPG stems (Figure 7E).

Consistent with other reports [7], we found ginsenoside Rf only in NZPG, and p-F11 only in NZPQ. They can be used as a reference marker to differentiate between Asian ginseng and American ginseng. We need to note that the two ginsenosides have very similar retention times in HPLC, and often co-elute. Thus, they are easily confused if identifications are made only according to the retention time of compounds. However, ginsenosides Rf and p-F11 can produce unique ions at *m/z* (637, 475) and (653, 491) in negative MS^2^ spectrum, respectively, due to fragmentation differences in their sugar moieties. Therefore, LC–MS^2^ can be an effective and sensitive method to identify ginsenoside Rf and p-F11 and further distinguish Asian ginseng and American ginseng, even without reference standards.

The literature reports that the contents of ginsenosides Rg1, Rb2, and Rc in Asian ginseng are higher than they are in American ginseng, and in Asian ginseng, the contents of Rb1, Re, and Rd are lower than they are in American ginseng [4]. In this study, we found that the contents of Rg1, Rb2, and Rc in NZPG are higher than they are in NZPQ only in the main root, and the contents of Rb1, Re, and Rd in NZPG are lower compared to the contents in NZPQ. However, in other parts, this rule has not been maintained. A few ginsenosides in the fine root (Rb2, Rd) and rhizome (Rg1, Re) do not comply with this rule, while, particularly in the leaf, the contents of these ginsenosides in NZPG are higher than that in NZPQ. A recent publication found the ratios of Rg1/Re < 0.15, Rb1/Rg1 > 2.15, and Rb2/Rc < 0.26 as the ginsenoside markers of American ginseng (as opposed to Asian ginseng) [9]. In the main root of New Zealand-grown ginseng, we found that Rb1/Rg1 > 2.15 and Rb2/Rc < 0.26 are in American ginseng and Rb1/Rg1 < 2.15, Rb2/Rc > 0.26, and Rg1/Re > 0.15 are in Asian ginseng, which is in line with the published report. In the rhizome, the ratios of these ginsenosides are similar between the two ginseng species. However, in other tissues, these ratios are waved and do not exhibit an obvious rule. This may be due to the uninterrupted interaction between the fine roots (root hair) and soil (microorganism), and the leaves and atmosphere (solar energy) leading to variation in ginsenosides in these parts. The transport of ginsenosides from various tissue parts needs to be explored in future experiments. Moreover, ginseng is typically grown in a farm setting under artificial shade conditions often involving pesticides and fertilizers [20,21], and managed by unified practice. Whereas this ginseng is certified organic grown under the shade of a pine forest, accumulation of ginsenosides can be affected by some uncontrolled factors, including soil, moisture, and light [22].

## 4. Conclusions

**Figure 8:**
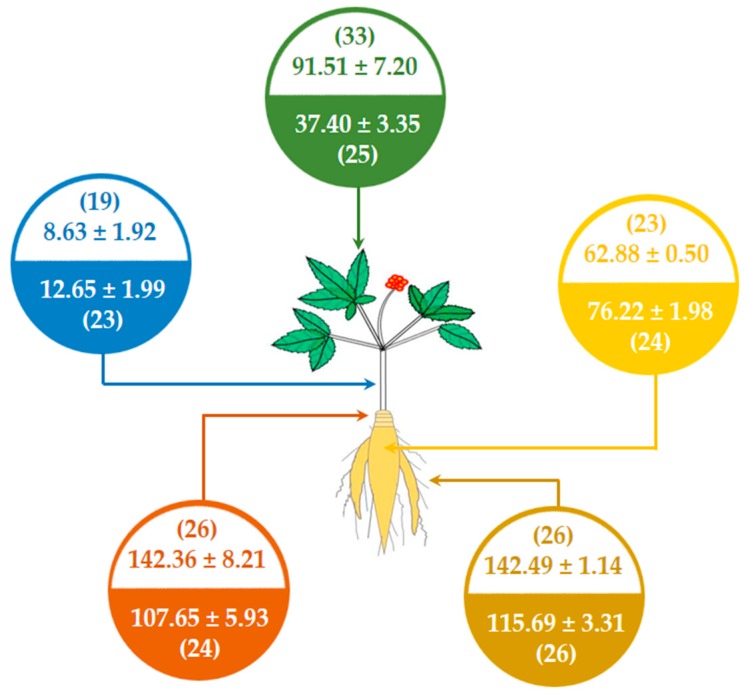
Comparison of ginsenoside components between New Zealand-grown Asian ginseng (colorless background) and New Zealand-grown American ginseng (colored background) in leaf (green), stem (blue), rhizome (red), fine root (brown), and main root (yellow). The numbers in parentheses refer to the number of ginsenosides identified by qualitative analysis, and the other numbers refer to the total content of ginsenosides (mg/g) quantified by quantitative analysis.

This study systematically investigated the composition of ginsenosides in various tissues of NZPG and NZPQ through qualitative and quantitative analysis. The number of ginsenosides and the total contents of ginsenosides in different parts between two ginseng variations is summarized in Figure 8. The ginsenoside composition and content of the two kinds of ginseng are very different in the aboveground parts (leaf and stem), and in the underground parts (main root, fine root, and rhizome); Asian ginseng and American ginseng have relatively similar ginsenoside constituent in the number of ginsenosides as well as the total ginsenoside amounts.

## Figures and Tables

**Table 1:** The ginsenosides identified from various parts of New Zealand-grown *Panax ginseng* (PG) and *Panax quinquefolium* L. (PQ).

No.	R.t	Measured Value [ion form]	Formula	Source	Identification	Ref.
1	3.50	815.4775 [M - H]; 861.4822 [M + HCOO]	C_42_H_72_O_15_	PG (m)	Ginsenjilinol	[15]
2	4.71	961.5291 [M - H]; 1007.5387 [M + HCOO]	C_48_H_82_O_19_	PG (m, r)	20glc-Rf	[11]
3	5.09	931.5215 [M - H]; 977.5257 [M + HCOO]	C_47_H_80_O_18_	PG (l); PQ (l)	NG-R1	[11]
4	5.62	931.5162 [M - H]; 977.5212 [M + HCOO]	C_47_H_80_O_18_	PG (f, r, m, l)	Re4	[16]
5	8.19	799.4857 [M - H]; 845.4898 [M + HCOO]	C_42_H_72_O_14_	PG (f, r, m, s, l); PQ (f, r, m, s, l)	Rg1	std
6	8.55	945.5370 [M - H]; 991.5439 [M + HCOO]	C_48_H_82_O_18_	PG (f, r, m, s, l); PQ (f, r, m, s, l)	Re	std
7	11.49	799.4835 [M - H]; 845.4920 [M + HCOO]	C_42_H_72_O_14_	PQ (s, l)	Rg1 isomer	[16]
8	11.53	841.4871 [M - H]	C_44_H_74_O_15_	PG (f); PQ (f)	Ac-Rg1	[16]
9	11.70	885.4780 [M - H]	C_45_H_74_O_17_	PG (m, r); PQ (m, r)	m-Rg1	[11]
10	13.17	1031.5386 [M - H]	C_51_H_84_O_21_	PG (f, r, m, s, l)	m-Re	[11]
11	13.83	799.4821 [M - H]; 845.4770 [M + HCOO]	C_42_H_72_O_14_	PQ (l)	Rg1 isomer	[16]
12	14.14	887.4954 [M + HCOO]	C_44_H_74_O_15_	PQ (f, m, r)	Yesanchinoside D	[16]
13	14.12	815.4697 [M - H]	C_42_H_72_O_15_	PG (l)	Re5	[15]
14	14.81	961.5322 [M - H]; 1007.5338 [M + HCOO]	C_48_H_82_O_19_	PG (l)	VG-R4	[17]
15	15.55	829.4839 [M + HCOO]	C_42_H_72_O_13_	PG (l)	G-La	[18]
16	15.88	1117.5382 [M - H]	C_54_H_86_O_24_	PG (r)	mf-Rd6/isomer	[6]
17	16.40	799.4807 [M - H]; 845.4855 [M + HCOO]	C_42_H_72_O_14_	PG (f, r, m, s, l)	Rf	std
18	16.55	799.4772 [M - H]; 845.4862 [M + HCOO]	C_42_H_72_O_14_	PQ (f, r, m, s, l)	p-F11	std
19	16.84	1325.6255 [M - H]	C_62_H_102_O_30_	PG (f)	m-Ra3/m-NG-R4	[6]
20	16.84	653.3698 [M - H]; 699.4271 [M + HCOO]	C_36_H_62_O_10_	PQ (f)	G-Ki/G-Km/G-ST2	[6]
21	17.09	799.4497 [M - H]; 845.4817 [M + HCOO]	C_42_H_72_O_14_	PG (l)	Rf isomer	[16]
22	17.40	769.4681 [M - H]; 815.4726 [M + HCOO]	C_41_H_70_O_13_	PG (f, r, m, s, l)	NG-R2	[11]
23	17.82	1209.6224 [M - H]	C_58_H_98_O_26_	PG (f, r, m)	Ra1	[11]
24	17.90	769.4739 [M - H]; 815.4754 [M + HCOO]	C_41_H_70_O_13_	PG (s, l)	G-F5	[17]
25	18.02	1239.6273 [M - H]	C_59_H_100_O_27_	PG (f)	Ra3/NG-R4	[17]
26	18.22	1107.5929 [M - H]	C_54_H_92_O_23_	PG (f, r, m); PQ (f, r, m, s)	Rb1	std
27	18.61	769.4658 [M - H]; 815.4750 [M + HCOO]	C_41_H_70_O_13_	PG (l)	G-F3	[17]
28	18.66	783.4858 [M - H]; 829.4930 [M + HCOO]	C_42_H_72_O_13_	PG (f, r, m, s); PQ (f, m, s, l)	Rg2	std
29	19.04	1193.5932 [M - H]	C_57_H_94_O_26_	PG (f, r, m); PQ (f, r, m, s)	m-Rb1	[11]
30	19.05	683.4329 [M + HCOO]	C_36_H_62_O_9_	PG (s); PQ (l)	Rh1	std
31	19.31	1077.5818 [M - H]	C_53_H_90_O_22_	PG (f, r, m, l); PQ (f, r, m)	Rc	std
32	19.49	1209.6254 [M - H]	C_58_H_98_O_26_	PG (f, r, m)	Ra2	[11]
33	19.75	1193.5849 [M - H]	C_57_H_94_O_26_	PQ (f, m)	m-Rb1 isomer	[16]
34	20.01	955.4865 [M - H]	C_48_H_76_O_19_	PG (f, r, m, s, l); PQ (f, r, m, s)	Ro	[11]
35	20.38	1163.5798 [M - H]	C_56_H_92_O_25_	PG (f, r, m, l); PQ (f, r, m)	m-Rc	[16]
36	20.80	1077.5813 [M - H]	C_53_H_90_O_22_	PG (f, r, m, s, l); PQ (f, m, s, l)	Rb2	std
37	20.98	955.4853 [M - H]	C_48_H_76_O_19_	PQ (r, s)	Ro isomer	[16]
38	20.95	1193.5892 [M - H]	C_57_H_94_O_26_	PQ (m)	m-Rb1 isomer	[16]
39	21.35	1077.5782 [M - H]	C_53_H_90_O_22_	PG (f, r, m, l); PQ (f, r, m, s, l)	Rb3	std
40	21.95	1163.5767 [M - H]	C_56_H_92_O_25_	PG (f, r, m, s, l); PQ (f, r, m, s, l)	m-Rb2	[11]
41	22.59	1163.5782 [M - H]	C_56_H_92_O_25_	PG (f, r, m); PQ (f, r, m, s, l)	m-Rb3	[11]
42	22.69	637.4292 [M - H]; 683.4310 [M + HCOO]	C_36_H_62_O_9_	PG (s, l)	F1	[17]
43	23.56	1163.5822 [M - H]	C_56_H_92_O_25_	PQ (s, l)	m-Rb3 isomer	[16]
44	24.65	945.5364 [M - H]; 991.5469 [M + HCOO]	C_48_H_82_O_18_	PG (f, r, m, s, l); PQ (f, r, m, s, l)	Rd	std
45	24.59	1119.5871 [M - H]	C_55_H_92_O_23_	PG (r)	Rs1	[19]
46	24.99	793.4306 [M - H]	C_42_H_66_O_14_	PQ (r, s)	Zingibroside R1	[11]
47	25.84	1119.5844 [M - H]	C_55_H_92_O_23_	PQ (l)	Rs2	[19]
48	26.35	1031.5348 [M - H]	C_51_H_84_O_21_	PG (f, r, m, s, l); PQ (f, r, m, s, l)	m-Rd	[11]
49	27.36	987.5413 [M - H]	C_50_H_84_O_19_	PG (s, l); PQ (f, r, s)	Ac-Rd	[18]
50	27.76	1031.5348 [M - H]	C_51_H_84_O_21_	PG (l); PQ (s, l)	m-Rd isomer	[16]
51	28.41	945.5354 [M - H]; 991.5435 [M + HCOO]	C_48_H_82_O_18_	PG (l); PQ (f, r, m, l)	GyXVII	[17]
52	29.12	1117.5369 [M - H]	C_54_H_86_O_24_	PG (s, l); PQ (s)	mf-Rd6 isomer	[6]
53	29.56	987.5449 [M - H]	C_50_H_84_O_19_	PG (f); PQ (f, r, m)	Ac-Rd isomer	[16]
54	29.63	1117.5314 [M - H]	C_54_H_86_O_24_	PQ (s, l)	mf-Rd6 isomer	[6]
55	29.67	987.5436 [M - H]	C_50_H_84_O_19_	PG (s, l)	Ac-Rd isomer	[16]
56	30.10	915.5285 [M - H]; 961.5338 [M + HCOO]	C_47_H_80_O_17_	PG (f, r, m, l); PQ (f, m, l)	NG-Fe	[17]
57	30.42	915.5226 [M - H]; 961.5279 [M + HCOO]	C_47_H_80_O_17_	PG (f, l); PQ (f, l)	VG-R16	[16]
58	30.75	1001.5258 [M + HCOO]	C_48_H_76_O_19_	PQ (l)	Ro isomer	[16]
59	30.91	829.4906 [M + HCOO]	C_42_H_72_O_13_	PG (s, l); PQ (f, m, l)	F2	std
60	31.15	793.4334 [M - H]	C_42_H_66_O_14_	PG (r, s); PQ (f, m, r, s)	Chikusetsusaponin IVa	[11]
61	31.4	783.4806 [M - H]; 829.4874 [M + HCOO]	C_42_H_72_O_13_	PG (l); PQ (l)	Rg3	std
62	31.43	793.4301 [M - H]	C_42_H_66_O_14_	PQ (r, s)	Chikusetsusaponin IVa isomer	[16]
63	31.43	825.4879 [M - H]; 871.4974 [M + HCOO]	C_52_H_72_O_11_	PQ (l)	Unknown	-
64	31.65	675.3529 [M - H]; 721.3579 [M + HCOO]	C_40_H_54_O_9_	PG (s, l)	Unknown	-
65	31.83	675.3505 [M - H]; 721.3589 [M + HCOO]	C_40_H_54_O_9_	PG (s, l); PQ (s, l)	Unknown	-
66	32.23	677.3731 [M - H]; 723.3730 [M + HCOO]	C_40_H_54_O_9_	PG (m); PQ (m)	Unknown	-
67	32.46	677.3743 [M - H]; 723.3746 [M + HCOO]	C_40_H_54_O_9_	PG (m); PQ (m)	Unknown	-
68	32.69	653.3673 [M - H]; 699.3744 [M + HCOO]	C_36_H_62_O_10_	PG (l); PQ (l)	G-Ki/G-Km/G-ST2	[6]
69	33.05	513.2942 [M - H]; 559.3044 [M + HCOO]	C_27_H_46_O_9_	PG (l); PQ (s, l)	Unknown	-
70	33.42	667.4373 [M + HCOO]	C_36_H_62_O_8_	PG (l)	Rh2	std
71	33.81	595.2873 [M + HCOO]	C_33_H_42_O_7_	PG (r, m, s); PQ (r, m)	Unknown	-
72	34.05	515.9695 [M - H]; 561.3247 [M + HCOO]	C_27_H_48_O_9_	PG (m)	Unknown	-

Note: f, r, m, s, and l refer to fine root, rhizome, main root, stem, and leaf of ginseng, respectively.

## Supplementary Materials

The following are available online at https://www.mdpi.com/2218-273X/10/3/372/s1: Table S1: The regression equations, linear ranges, limits of detection and limits of quantification of 14 ginsenosides.
