# Supplementary material for: Comparison of Ginsenoside Components of Various Tissues of New Zealand Forest-Grown Asian Ginseng (Panax Ginseng) and American Ginseng (Panax Quinquefolium L.)
Source: Biomolecules. 2020 Feb 28;10(3):372. doi: 10.3390/biom10030372 (PMC7175180; doi:10.3390/biom10030372)
Supplement: Supplementary file 1 [file biomolecules-10-00372-s001.pdf]

Supplementary data

Table S1. The regression equations, linear ranges, limits of detection and limits of quantification of 14 ginsenosides

Ginsenosides	Calibration curve	r^2	Concentration range (mg/L)	LOD (mg/L)	LOQ (mg/L)
Rb1	y=40414x+29985	0.9986	2.00-128.16	2.07	6.91
Rb2	y=42370x-36168	0.9994	2.20-141.00	2.06	6.85
Rb3	y=41463x-24949	0.9998	1.80-57.66	1.10	3.68
Rc	y=36973x-30003	0.9995	1.87-119.68	1.64	5.48
Rd	y=76766x+18313	0.9988	2.10-67.28	2.86	9.52
Re	y=74382x-12033	0.9999	2.40-153.84	0.89	2.97
Rf	y=68030x+39879	0.9996	2.54-162.44	2.04	6.79
p-F11	y=153686x+46754	0.9998	3.61-57.69	1.01	3.37
F2	y=114456x+3865.7	0.9995	2.40-38.44	1.17	3.89
Rg1	y=117288x+46652	0.9994	3.01-192.32	2.84	9.46
Rg2	y=142443x+52824	0.9997	1.60-102.52	1.03	3.44
Rg3	y=100975x+2937.7	0.9998	2.00-32.06	0.59	1.96
Rh1	y=161372x+41690	0.9994	1.74-27.78	0.87	2.89
Rh2	y=194885x-27449	0.9999	2.34-18.70	0.31	1.04

y is the integrated peak area and x is the amount of analyte (ng). LOD: limit of detection; LOQ: limit of quantification.
